# The Musculoskeletal Manifestations of COVID-19: A Narrative Review Article

**DOI:** 10.7759/cureus.29076

**Published:** 2022-09-12

**Authors:** Alan J Alexander, Abhishek Joshi, Ashok Mehendale

**Affiliations:** 1 Epidemiology and Public Health, Jawaharlal Nehru Medical College, Datta Meghe Institute of Medical Sciences, Wardha, IND; 2 Community Medicine, Jawaharlal Nehru Medical College, Datta Meghe Institute of Medical Sciences, Wardha, IND

**Keywords:** sars-cov-2, arthralgia, myalgia, musculoskeletal manifestations, covid-19

## Abstract

The coronavirus pandemic has caused a devastating impact across the planet. Millions of lives lost and economic structures are struggling to remain afloat. Clinical effects of SARS CoV-2 virus include tiredness, fatigue, headache, cough, loss of appetite, fever, loss of sensations of taste, and smell as well as other respiratory difficulties. Pulmonary complications of coronavirus infections result in severe pneumonia with the final sequelae being sepsis, and end-stage respiratory failure. Further cardiovascular, neurological, hematological, and gastrointestinal complications build up to cause the demise of the immune system ultimately leading to death of the affected individual. The attack of the virus and the resultant reaction of the epithelial cells lining the respiratory tract have been in the limelight of most studies pertaining to the pandemic. However, a lesser number of studies have detailed the muscular and osseous pathologies that appear post-coronavirus infection. Inflammation post-infection, across the organ systems, may appear as a link to bone and joint pathology. Myalgia is a typical COVID-19 infection symptom. On the contrary, other musculoskeletal signs have very seldom been reported. Multimodality imaging techniques stand a chance at showing the diagnosis and the degree of follow-up after evaluation. Apart from myalgia, there are cases of arthralgia, myopathies, and neuropathies. According to numerous reports, there is the possibility of a link between the current drug regimen used to treat the SARS-CoV-2 infection and the musculoskeletal manifestations observed. In this study, we aim to shed light on the coronavirus pandemic and its association to various musculoskeletal manifestations, provide a different perspective of the infected patients, and address the major points that a clinician must take care while administering care to the patient. We will also address the present treatment in line with the various musculoskeletal symptoms observed.

## Introduction and background

The coronavirus pandemic is a coming-of-age infectious respiratory disease. The disease is caused by the novel severe acute respiratory syndrome coronavirus 2 (SARS-CoV-2). It was first observed and documented in the city of Wuhan located in the province of Hubei in Central China. The coronavirus disease 2019 (COVID-19) was officially declared a global pandemic on March 11, 2020, by the World Health Organization. At this date, the coronavirus had spread across 114 countries and affected more than 118,000 individuals across the world, resulting in enormous fatalities [1]. Fast forward two years ahead, and the global number of cases stand at a deadly figure of 455 million with over 6 million fatalities [2]. Risk factors include comorbidities such as cardiovascular diseases, diabetes mellitus, old age, obesity including hypertension. Coronavirus can be divided into five groups on the basis of the severity of the illness [3]. These include asymptomatic or pre-symptomatic infection followed by mild illness, moderate illness, severe illness, and the most dangerous of them all, critical illness.

Asymptomatic coronavirus cases, together with mild coronavirus cases, account for most of the cases. These cases have hallmark symptoms which are fever, loss of taste sensation as well as smell, malaise, shortness of breath, gastrointestinal (GI) distress, and headaches. Patients rarely present with mild pneumonia which results in the hesitation to proceed or not for treatment. Severe COVID-19 patients need immediate hospitalization to treat the infection due to depression of the respiratory system, and critical patients who experience respiratory failure (acute respiratory distress syndrome) require mechanical ventilation with high-flow oxygen support. Currently employed therapeutic options include COVID-19 vaccines (Pfizer-BioNTech, Moderna, Covaxin), antivirals (remdesivir and molnupiravir), Janus kinase inhibitors (baricitinib), convalescent plasma therapy, corticosteroids (dexamethasone), anticoagulants (heparin), cell therapy, monoclonal antibodies (bebtelovimab), and intravenous immunoglobulin therapy. Despite the fact that COVID-19 predominantly affects the respiratory system, multiple extra-pulmonary manifestations have been observed such as kidney injury, GI symptoms, liver injury, myocardial ischemia, acute coronary syndromes, and neurological manifestations with various dermatological symptomatic presentations [4-6].

Myalgia is yet another clinical feature of the coronavirus, although cases of musculoskeletal manifestations have been uncommonly described early in the coronavirus pandemic. As the global spread of the pandemic continued, the number of both patients and survivors rose and so did the increasing case reports of various musculoskeletal and rheumatological complications with regards to both SARS-CoV-2 and its treatment/course in the hospital [7]. Through this article, we aim to deliberate the various musculoskeletal manifestations of the coronavirus specifically at the muscles, bones, joints, and soft tissue.

## Review

The severe acute respiratory syndrome coronavirus 2 (SARS-CoV-2)

The Coronaviridae family hosts the ribonucleic acid (RNA) virus known as SARS-COV-2. The positive sense RNA virus is single-stranded. It has a viral structural S protein (spike) which combines with the angiotensin-converting enzyme 2 (ACE 2) receptor of human cells [4,6]. Lung epithelial cells have the highest expression of ACE 2 receptors and less in the kidney, spleen, pancreas, and heart. The bladder, GI system, cornea, and other blood vessels also have decreased amounts of ACE 2 receptors. The single-strand virus enters the cell using the serine protease transmembrane protease, serine 2 (TMPRSS2) [8].

Proteolytic cleavage, post-virus-receptor binding, of spike (S) protein due to TMPRRS2 reveals a fusion peptide signal. This allows the blending of both human and viral membranes which facilitates the viral RNA release into the human cell cytoplasm [9]. The translation and replication of the viral RNA then take place. This ultimately leads to the virions assembling to be liberated from the affected cells by the process of “exocytosis” [9]. Coronavirus-targeted proteins are involved in inflammatory signaling, ubiquitin ligases, mitochondrial respiration, and cytoskeletal stability. As the viral load of cells increases so does the risk of disruption of fundamental cellular functions which eventually leads to apoptosis [10]. The apoptotic cells eventually chip in to “tissue level dysfunction” and amplify local level inflammation. If we include SARS-CoV-2, six other strains of the coronavirus are now known that are transmissible to humans. This includes four strains that are less severe which result in symptoms that are mild, and the more severe viruses with increased pathogenicity, i.e., SARS-CoV-1, responsible for “severe acute respiratory syndrome” (SARS), & MERS CoV, responsible for the “Middle East respiratory syndrome” [11]. It’s been observed that there is a high resemblance between the sequences of the genes of SARS-COV-2 as well as SARS-COV-1. In addition to this, a huge similarity exists in the postulated viral-human interactome between the two strains.

Biochemistry of viral cell interaction

Coronavirus is believed to primarily affect type-II pneumocytes in the epithelial lining of the respiratory tract, which expresses TMPRSS217 and ACE 2 [12]. However, viremia can be observed in coronavirus-infected individuals due to the epithelium of the alveoli that has been significantly damaged [13]. Thus, it can be reasoned to believe that the infection can spread to other cells in the body. Macrophages, mast cells, type 1 as well as type 2 alveolar cells, and B and T cells all exhibit TMPRSS2 and ACE2 within the epithelial lining of the respiratory airway. The TMPRSS2 is expressed by a variety of cell types in human skeletal muscle tissue, but only smooth muscle and pericytes express the ACE2. Fibroblasts, B cells, T cells, and monocytes are among the synovium’s other cells that express TMPRSS2 as well as ACE2. Only homeostatic chondrocytes express TMPRSS2, while hypertrophic, proliferative as well as effector chondrocytes express ACE2 in articular cartilage. A tiny percentage of regulatory fibrochondrocytes and cartilage progenitors of the meniscus express just ACE2, not TMPRSS2. In composite bone tissue, TMPRSS2 is nearly undetectable. Direct coronavirus infection has the ability to infect cortical bone, synovium, and skeletal muscle.

Muscle involvement in SARS-CoV-2 infection

Myalgia is primarily defined as muscle aches and pain. It can involve the ligaments, and soft tissues which connect muscles, tendons and fascia, bones, and organs. Causative factors include trauma, overuse, tensions, certain pharmaceutical drugs, and various other ailments. Myalgia has been reported in several cohorts of patients with COVID-19 infection [14].

Multiple cases have detailed rhabdomyolysis and myositis in coronavirus patients as a presenting symptoms as well as a late complication. There are reports of SARS-CoV-2-induced “necrotizing autoimmune myositis” in a few cases [6]. The mechanisms behind COVID-19’s muscle involvement however are unknown. SARS-CoV-2 has been linked to hematogenous dissemination and direct skeletal muscle invasion via the ACE2 receptor [5,6]. Immune-mediated pathways, which occur subsequent to an inflammatory response coupled with hypercytokinemia and immune cell activation, are a more widely recognized explanation of SARS-CoV-2 muscle involvement. Cytokine releases that are proven to be myotoxic, deposition of immune complexes, and damage caused by the extensive similarity between viral antigens and human muscle cells have all been proposed as pathways for immune-mediated muscle damage [5,6]. Myositis and rhabdomyolysis have been seen as presenting symptoms as well as delayed complications in COVID patients. SARS-CoV-2-induced necrotizing autoimmune myositis has also been reported in a few cases [15]. The mechanisms behind COVID-19’s muscle involvement are still under investigation. SARS-CoV-2 has been linked to blood-borne dissemination and direct skeletal muscle invasion via the ACE2 receptor [7]. Myositis is a general term used for muscle inflammation that has been observed in coronavirus infection and other illnesses caused by viruses such as human immunodeficiency virus (HIV), hepatitis virus, and human Influenza A and B virus [16]. On the other hand, rhabdomyolysis is a myositis complication characterized by muscular infarction and elevated myoglobin amounts in the blood. This is potentially a fatal illness as it can result in disseminated intravascular coagulation (DIC), compartment syndrome, and acute renal failure [17]. Common clinical signs observed are myalgia and raised creatine phosphokinase levels, both seen in coronavirus patients having myositis or rhabdomyolysis.

The gold standard for diagnosis is a muscle biopsy. It can be supported by imaging and delineated locations for muscle biopsy [5]. The modality of choice is MR imaging, particularly with a 1.5-T or higher field strength. Disease patterns observed are hyperintense signals that are homogeneous and enhanced, known as type 1 followed by type 2 which are also hyperintense signals but are heterogeneous and show enhancement at the rim [5]. Sites of necrosis and absence of normal muscle architecture can be visible in severe illness. The “stipple sign” is a defining feature of myonecrosis. It is seen as non-enhancing muscle tissue having a rim-enhanced area with a focus of enhancement within areas of necrosis.

Critical illness myopathy, a prevalent acquired syndrome in ICU patients requiring ICU treatment, is a differential diagnosis for muscular edema on MRI in hospitalized COVID-19 patients [15]. It’s also been connected to the use of corticosteroids [18]. Acute flaccid quadriplegia or symmetric and widespread weakness are common symptoms of critical disease myopathy [15]. A primary myopathy with non-specific imaging indications of multifocal muscular edema and atrophy is known as critical illness myopathy [18]. Cachexia and sarcopenia are both long-term muscle sequelae of COVID-19 [14]. Reduced muscle size and fat infiltration are MRI findings in muscular atrophy, as seen in cachexia as well as sarcopenia [14].

Nerve involvement in SARS-CoV-2 infection

Peripheral neuropathy is nowadays increasingly being documented in cases of coronavirus [19]. The pathways of neural involvement of the coronavirus are still under investigation.

SARS-CoV-2 virus proteins were revealed in the medulla oblongata as well as cranial nerves 9 and 10, in an established series of postmortems performed on COVID-19 patients [20]. The SARS-CoV-2 virus has the potential to be a novel neuropathogenic virus by targeting, affecting, and invading peripheral nerves through the ACE-2 receptors should be investigated further [21]. Molecular mimicry can also be suggested as a possible hypothesis that could explain peripheral nerve injury [18]. Another possible explanation is that the virus has direct cytotoxic effects on peripheral neurons [8].

Several studies of the Guillain Barre syndrome (GBS) following the onset of the coronavirus infection have been documented [22]. Just 3 to 4 weeks post the onset of the SARS-CoV-2 infection, the GBS symptoms appear. It is a demyelinating type of polyneuropathy that causes “acute ascending paralysis” in most people. Neuropathies in the brain and brain stem coupled with facial weakness are potential possible side effects. “Miller Fisher” syndrome, another GBS variation, is characterized by ophthalmoplegia, ataxia of gait, and areflexia. Miller Fisher syndrome has also been linked to COVID-19. The cauda equina and nerve roots/plexus show mild to moderate contrast enhancement along with signal hyperintensity and enlargement on MR imaging in GBS and GBS variations.

Iatrogenic peripheral neuropathy has been described with an increased incidence in view of the coronavirus pandemic. The SARS-CoV-2-infected individuals can be at an increased risk of iatrogenic nerve damage attributed to the hyperinflammation caused by the coronavirus. It may also be that the overlapping comorbidities can contribute to severe COVID-19 symptoms as well as neural injuries, thus requiring immediate hospitalization [22]. Maximum utilization of oxygen in hospital-admitted COVID patients is of utmost priority. To facilitate this, the position of proning is routinely done. Paradoxically these approaches might cause negative reactions in the peripheral nervous system [23]. Injuries of the peripheral nerves such as stretch/traction injuries may take place due to repositioning maneuvers and prone positioning while in the hospital. If the patient is in a prolonged state of prone positioning, it can result in external compression. An intramuscular hematoma will result in internal compression. Both these states can cause peripheral nerve compression injury. Critical illness polyneuropathy has been reported to be linked to COVID-19 ICU stay [24]. Critical illness polyneuropathy is often bilaterally symmetric. However in former ICU patients, non-symmetric involvement of a myotome or a peripheral nerve dermatome is the earliest sign of neuropathy related to positioning. If hematoma-related compression neuropathy is the case, segmental nerve constriction due to mass effect might be detected.

Joint involvement in SARS-CoV-2 infection

Coronaviruses are more commonly linked to arthralgia and myalgia as compared to clinical arthritis [25]. Arthralgia is now identified to be a COVID-19 symptom. A single study has proved 2.5% of patients clinically experiencing it, hence distinguishing arthralgia from myalgia as a separate symptom [26]. The number of cases with respect to acute clinical arthritis are relatively few to date that are reported. Some of these cases have hallmarks suggestive of reactive arthritis as compared to viral arthritis. It hence emphasizes the importance of ruling out other causes of arthropathy in these cases [27,28]. A case of viral-induced arthritis is difficult to diagnose, despite the fact that symptoms such as arthralgia post a couple of weeks of infection, a course that resolves on its own as well as satisfactory responses toward non-steroidal anti-inflammatory drugs (NSAIDs) are all signs of viral arthritis. Systemic lupus erythematosus (SLE), rheumatoid arthritis, Graves’ disease, dermatomyositis as well as psoriatic spondyloarthritis are among the chronic rheumatologic disorders caused by SARS-CoV-2 [29-33]. In infected individuals having minor or none of the respiratory symptoms during the acute viral infection, SARS-CoV-2 can cause inflammatory arthropathies [34]. Hence there is a need to correlate to the coronavirus testing to prove this link.

Bone involvement in SARS-CoV-2 infection

At the present moment, data with respect to the osseous complications of the coronavirus is relatively less. Osteoporosis followed by osteonecrosis may develop due to critical illness, virus-induced coagulopathy, and even corticosteroid therapy [35]. The advantages and benefits of steroid therapy have been established through the REMAP-CAP and RECOVERY clinical trials [36,37]. On radiography, osteonecrosis may present with osteosclerotic changes and on CT as low signal serpiginous lines. The MRI however would present osteonecrosis with a central hyperintense line. Later on, adjacent bone marrow would show edema patterns. An articular surface collapse may also be present [38].

Soft tissue involvement in SARS-CoV-2 infection

Severe cases of COVID-19 lead to DIC due to bleeding and thrombotic manifestations [39]. Gangrene is frequently illustrated in patients that can be attributed to medications given for hemodynamic support or due to thrombotic manifestations of the SARS-CoV-2 [40]. Patients with underlying comorbidities such as diabetes or peripheral vascular diseases can cause distal extremities to be at greater risk of gangrene. Multi-modular imaging depicts ulcerations on the skin, T2 signal hypersensitivity of the soft tissues, and devitalized tissue’s lack of enhancement. CT identifies “wet” gangrene as soft-tissue gas. Subcutaneous soft tissues and muscles have an increased risk of the development of hematomas [38]. They give rise to serious complications such as superimposed infection or compressive neuropathy. Risk of increased compartment pressure is also to be noted.

## Conclusions

As each day brings new hope for a cure and fears over a reemergence of the coronavirus, the increased focus must be directed toward its musculoskeletal manifestations. COVID-19 can present with myalgias, arthralgias, myopathies, neuropathies, bone, and joint damage. Further light should be shed upon the musculoskeletal signs and symptoms that can be precipitated during the course of the COVID therapy. More benefits have to be reaped from steroid therapy.

Imaging techniques such as radiography, CT, and MRI play a key role in aiding the diagnostic evaluation of COVID-19-infected musculoskeletal pathologies. They can be used both for initial prognosis followed by an update of the therapy to examine the patient’s status through the course of the disease. Through all this, the peak need of the hour is the effective rehabilitation of recovered individuals. This is essential to aid the patients in returning to their previous physical states of mobility and function, well before their COVID-19 infection.
